# Comparative Phytochemical Analysis of *Aronia melanocarpa* L. Fruit Juices on Bulgarian Market

**DOI:** 10.3390/plants11131655

**Published:** 2022-06-22

**Authors:** Oskan Tasinov, Ivayla Dincheva, Ilian Badjakov, Christina Grupcheva, Bistra Galunska

**Affiliations:** 1 Department of Biochemistry, Molecular Medicine and Nutrigenomics, Faculty of Pharmacy, Medical University of Varna, 84B Tzar Osvoboditel Blvd., 9002 Varna, Bulgaria; oskan.tasinov@gmail.com; 2 AgroBioInstitute, Agricultural Academy, 8 Dr. Tsankov Blvd., 1164 Sofia, Bulgaria; ivadincheva@yahoo.com (I.D.); ibadjakov@gmail.com (I.B.); 3 Department of Ophthalmology and Visual Sciences, Faculty of Medicine, Medical University of Varna, 15 Doyran Street, 9000 Varna, Bulgaria; hristina.grupcheva@mu-varna.bg

**Keywords:** *Aronia melanocarpa*, fruit juice, phenolic acids, polyphenols, functional foods

## Abstract

*Aronia melanocarpa* L. (black chokeberry), belonging to the Rosaceae family, contains high amounts of polyphenolics and therefore exhibits one of the highest antioxidant and anti-inflammatory activities among berry fruits. Chokeberries are used in the food industry for juice, nectar, and wine production and as colorants. We aimed to compare the phytochemical composition of three chokeberry juices commercially available in the local market as sources of beneficial phytochemicals. Using GC–MS and LC–MS/MS, we performed the identification and quantitation of polar compounds and polyphenolics. The concentrations of 13 amino acids, including 6 essential amino acids, 10 organic acids, 20 sugar alcohols and derivatives, 14 saccharides, 12 fatty acids and esters, and 38 polyphenols, were estimated. One of the analyzed juices had the highest polyphenolic content (5273.87 ± 63.16 µg/mL), possibly due to 2.9 times higher anthocyanin concentration compared to anthocyanins in other tested juices. This study provides new data concerning phytochemical composition in terms of amino acids, organic acids, sugar acids, fatty acids and their esters, and polyphenols as phytocomponents of commercially available chokeberry juices. Results show that after all processing techniques and possibly different plant growth conditions, chokeberry juices are a valuable source of health-promoting phytochemicals such as phenolic acids, pro-anthocyanins, and anthocyanins, thus considering them as functional foods. We demonstrated a diversity of the active substances in bioactive foods marketed as “same”; therefore, the standardized therapeutic effect could be expected only by the utilization of food supplements with guaranteed constant content.

## 1. Introduction

*Aronia melanocarpa* L. (AM, black chokeberry) belongs to the Rosaceae family, subfamily Maloideae, and is native to the eastern parts of North America shrub, growing to a height of 2–3 m and forming purplish to black berries. It was transferred to Europe at the beginning of the 20th century and had recently been cultivated mainly in eastern European countries and Germany [1]. Chokeberry fruits are the most used part of the plant [2]. They are commonly used in the European food industry for the production of syrups, juices, jellies, nectars, wines, fruit teas, and dietary supplements [3,4,5]. In addition, the high content of anthocyanins in chokeberries suggests their usage as natural food colorants [6,7,8].

Chokeberries are used in North American traditional medicine as astringents and as a remedy for cold treatment. In Russia and Eastern European countries, they are mostly known as natural antihypertensive and anti-atherosclerotic remedies for the treatment of achlorhydria, avitaminoses, convalescence, and hemorrhoids [1,5,9,10,11].

*A. melanocarpa* L. fruits’ are rich in anthocyanins, flavonols, flavanols, proanthocyanidins, and phenolic acids [12]. Chokeberries are especially high in cyanidin glycosides, proanthocyanidins mono-, di-, and trimers, and hydroxycinnamic acids such as chlorogenic acids and quercetin glycosides [13]. The high polyphenol content of fruits is related to their strong in vitro [14,15] and in vivo antioxidant activity, including modulation of antioxidant enzymes [16,17,18]. In addition, they exert anti-inflammatory activity by decreasing inflammatory cytokine production and improving lipid profile by reducing chylomicron, LDL, and triglyceride levels and increasing HDL [19,20,21,22]. Regular consumption of chokeberry juice reduces blood pressure in individuals with metabolic syndrome and mild hypercholesterolemia [21,23]. The health benefits of chokeberry juice are supported by scientific studies pointing to its hepatoprotective, gastroprotective, antidiabetic, and anticancer activities [24,25,26,27,28]. AM juice exerts in vitro bacteriostatic activity against *Staphylococcus aureus* and *Escherichia coli,* antiviral activity against influenza type A virus [9], and reduces urinary tract infections [29].

The nutritional contribution and main biological effects of chokeberry fruits as a potential functional food are associated predominantly with their phytochemical content and especially with the presence of chlorogenic acids, cyanidin glycosides, and quercetin derivatives [30]. Since the periods of ripening and the time of harvesting of ripe fruits may vary, it is expected that fruit juices harvested from different regions and produced by different companies may vary in their polyphenolic, sugar, organic, and sugar acids content and, as a consequence, in their biological activity [4].

In recent years, the interest in locally produced functional foods, including chokeberry fruit juices, has been growing. Therefore, it would be useful to provide the consumers with a scientific assessment and an overview of the content of bioactive phytocompounds from different chokeberry juices available on the local market.

Recently we aimed to perform a comparative phytochemical analysis of chokeberry juices produced locally and available on the Bulgarian food market. Thus, assessing their quality as a source of bioactive compounds and functional food with known beneficial health effects, a selection among the tested AM fruit juices would be made and recommend the juice with the highest quality in dietary guidelines.

## 2. Results

### 2.1. Phytochemical Content and Composition

Detailed phytochemical analyses of selected *Aronia melanocarpa* L. fruit juices revealed the presence of 13 amino acids (AA), 10 organic acids (OA), 20 sugar alcohols and derivatives, 14 saccharides, 12 saturated and unsaturated acids and esters, and 38 polyphenols including anthocyanins, proanthocyanidins, stilbenes, cyclohexanecarboxylic acid, hydroxycinnamic acids, and flavonol glycosides.

#### 2.1.1. Polar Compounds

Analyses of the AA content revealed 41.8% essential AA for all tested AM juices. Juice 3 stands out with the highest AA content (106.88 ± 1.56 µg/mL) vs. juice 1 (*p* < 0.01) and 2 (*p* < 0.001) (Table 1). In all analyzed AM drinks, the highest content was determined for L-proline (22% of AA), L-aspartic acid (17.94% of AA), and L-phenylalanine (11.23% of AA). L-phenylalanine represents 26.9% of all detected essential AA in all analyzed AM drinks (Table 1).

Among the polar OAs highest concentrations in all AM drinks, pyroglutamic acid (5-oxoproline) (30.39% of OA) and isocitric acid (16.37% of OA) were found (Table 1). The highest concentrations of total OA (129.96 ± 2.01 µg/mL) and each individual OA were found in juice 3, followed by juice 2 (124.91 ± 1.93 µg/mL), and juice 1 (118.55 ± 2.34 µg/mL).

The dominating sugar alcohols in all analyzed AM drinks are sorbitol and its derivative sorbitol 6-phosphate (34.9% of alcohols), followed by glycerol and glycerol 3-phosphate (20.3% of alcohols), and then arabinitol (13% of alcohols). The sugar content was highest for juice 3, followed by juice 2 and 1.

Dominating monosaccharides are galactose and its 6-phosphate derivative (17.3% of all analyzed saccharides), followed by glucose and its 6-phosphate derivative (15.99% of saccharides), and fructose and its 6-phosphate derivative (13.43% of saccharides) (Table 1). Sucrose (11.03% of saccharides) was the main disaccharide, and raffinose (9.37% of saccharides) was the main trisaccharide. Juice 3 was leading in saccharide content as well.

Octadecadienoic acid (11.86% of lipids) was with the highest content among all detected fatty acids, followed by octadecanoic acid (8.42% of lipids), while beta-sitosterol is the sterol, representing 11.54% of detected lipids (Table 1).

#### 2.1.2. Polyphenolic Content

It is well known that fruit juices of *A. melanocarpa* L. are rich in polyphenolic compounds, but data on the quantitative and qualitative composition of the polyphenols they contain are very limited. Highest concentration of polyphenolics was detected in juice 3 (5273.87 ± 63.16 µg/mL), followed by juice 2 (4460.53 ± 136.67 µg/mL), and juice 1 (4351.83 ± 75.38 µg/mL) (Table 2).

The most abundant anthocyanin was cyanidin-3-O-galactoside, comprising a minimum of 70% of anthocyanins in all analyzed samples. The content of cyanidin-3-O-galactoside was about 2.6 times higher in juice 3 compared to juice 2 and 1. Epicatechine, the major proanthocyanidin in all tested samples, exhibited the highest concentration in juice 2 (269.47 ± 17.35 µg/mL) (Table 2). The leading in proanthocyanidin polymer content was juice 3, containing 552.05 ± 5.49 µg/mL epicatechin dimers and 732.42 ± 7.29 µg/mL trimers. The only detected stilbene in the tested juices was trans-resveratrol-3-O-glucoside (from 44.37 ± 1.79 µg/mL in juice 3 to 39.80 ± 1.63 µg/mL in juice 1). Quinic acid was the only detected cyclohexanecarboxylic acid (from 84.95 ± 0.42 µg/mL to 81.74 ± 2.27 µg/mL, in juice 3 and 1, respectively). Dominating hydroxycinnamic acids in the tested AM fruit juices were neochlorogenic and chlorogenic acid, followed by 3-O-p-coumaroylquinic acid. The total amount of hydroxycinnamic acids in the analyzed juices represents 51.52%, 51.38%, and 45.35% for juices 1, 2, and 3, respectively (Table 2). Hyperoside was found to be the major flavonol (14.93% of flavonols) detected in all analyzed samples.

**Figure 1:**
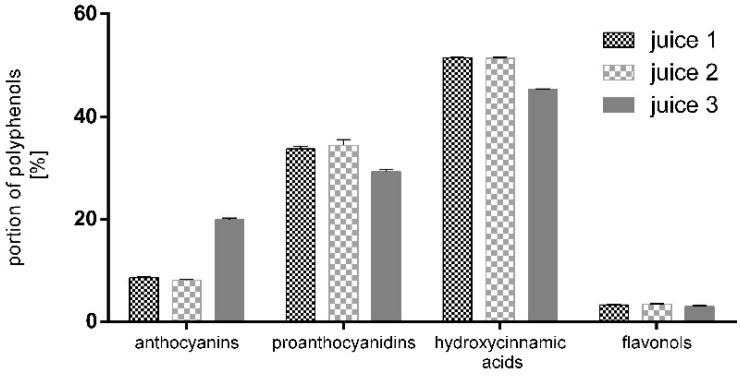
Content of anthocyanins, proanthocyanidins, hydroxycinnamic acids, and flavanols in the tested chokberry juices. Data are presented as % ± SD of the total polyphenolic content.

We compared the portions of different polyphenol classes within each of the analyzed juices (Figure 1). When calculated, the percentage of detected hydroxycinnamic acids varies from 45.3% to 51.5%, followed by the proanthocyanidins from 29.3% to 34.4%, anthocyanins from 8.1% to 19.9%, and flavonols from 3% to 3.3% as the lowest portion of identified polyphenols.

## 3. Discussion

As there are high variations in environmental and climate conditions, soil characteristics, maturation level, harvesting period, etc., the phytochemical composition and respective health benefits of chokeberries vary as well [31,32,33]. Recently, we have analyzed and compared the phytochemical composition and content of selected bioactive compounds in three *A. melanocarpa* L. fruit juices available on the Bulgarian market, produced by different local companies, and harvested from different regions. We have provided for the first time new qualitative and quantitative data regarding the phytochemical composition (AAs, Oas, sugar acids and alcohols, fatty acids and esters, and polyphenols) of commercially available chokeberry juices produced locally.

### 3.1. Amino Acids

Aas, especially essential ones, are valuable food components for living organisms. Literature data concerning the AA content of chokeberry juices are very scarce. There are data that AM fruits are rich in dry matter [34,35]; however, the amount of total protein is low [36]. The main amino acids, including essentials, found in chokeberry fruit pomace are glutamic and aspartic acid, arginine, tyrosine, histidine, leucine, lysine, cysteine, alanine, serine, and threonine [37]. We performed for the first time a comparative analysis of the amino acid content of three selected chokeberry juices from different local producers. The highest amino acid content, including essential AA, was found in juice 3 (106.88 ± 1.56 µg/mL). Thus, chokeberry fruit juice may be considered a natural source of AA.

### 3.2. Organic Acids

The OAs reported in chokeberries are tartaric, citric, isocitric, malic, succinic, fumaric, ascorbic, shikimic, and oxalic acids [33,38,39]. Literature data have shown the presence of citric, malic, oxalic, and tartaric acids in commercially available chokeberry juices [40]. In addition, we also detected succinic, isocitric, fumaric, pyroglutamic, 4-aminobutyric, 2-hydroxyglutaric, 2-ketoglutaric, phenylpyruvic, and 2.3-dihydroxybutanedioic acids in the tested chokeberry juices commercially available. It might be presumed that the content of pyroglutamic acid (5-oxoproline) in tested chokeberry juices may be due to the high content of its keto-derivative proline.

### 3.3. Saccharides, Sugar Acids and Alcohols

There are wide variations in the content of carbohydrates in chokeberry fresh fruits [12]. Monosaccharides reported by other authors in chokeberry juice are glucose and fructose [40]. In addition to glucose and fructose detected in all tested AM juices, we also found sugars such as galactose, sorbose, arabinose, and xylose. The main detected disaccharide in chokeberry juice was sucrose, as reported by other authors as well [29]. In our study, we also found the presence of trehalose and melibiose and raffinose, the main trisaccharide found for the first time in chokeberry juice.

There is only one study reporting the presence of galacturonic acid in chokeberry fruit pomace [41]. There are almost no data concerning the detailed sugar acid content in chokeberry fruit juices. We recently found the presence of glyceric, erithreonic, threonic, pentonic, ribonic, glucuronic, galacturonic, gluconic, galactonic, glucaric, and galactaric acid.

There are data that fruits [38] and juice [40] are considerably rich in sugar alcohols, mainly sorbitol. This finding was confirmed by our results as well, revealing that sorbitol is the main sugar alcohol in the tested samples (52.77 ± 1.04 µg/mL in juice 1; 57.86 ± 0.90 µg/mL in juice 3). In addition, we identified a few more sugar alcohols in chokeberry juice, including glycerol and arabinitol in higher concentrations and threitol, erythreol, xylitol, manitol, inositol, and galactitol in lower concentrations.

### 3.4. Fatty Acids and Esters

It was shown that *A. melanocarpa* fruits are rich in phospholipids, sterols, and α-tocopherols [42]. There are data that polyunsaturated fatty acids and especially linoleic acid are the main portions of fatty acids found in dried pomace and seeds of chokeberry [37,42]. The same study reports that β-sitosterol is the main sterol in chokeberry seeds, followed by campesterol and δ-avenasterol [42]. We have also detected β-sitosterol in chokeberry juice as well (Table 1). In our study, we reported for the first time the presence of fatty acids such as hexadecenoic, heptadecanoic, hexadecatrienoic, hexadecanoic (palmitic acid), octadecadienoic (linoleic acid), octadecanoic (stearic acid) acids, and esters as 1-monopalmitin, monooctadecanoyl glycerol (Table 1). The dominating fatty acids in the tested juice samples were the nonessential stearic acid and the essentials linoleic and linolenic acids. Health beneficial effects of β-sitosterol and essential fatty acids as vascular protectors and cholesterol-lowering agents are widely reported [43,44,45,46]. Considering recent findings regarding the fatty acid profile of chokeberry fruit juice, we may add new data explaining the potential of AM fruits and fruit juice in lipid profile improvement [19,21,22].

### 3.5. Phenolic Compounds

Plant-derived polyphenols are among the main bioactive compounds in our diet and comprise the main portion of antioxidants we intake. Their wide range of health benefits makes the proper intake of naturally derived foods and drinks rich in polyphenols an essential part of a healthy diet. Chokeberries have the highest polyphenolic content among different berries [47]. They are rich mostly in proanthocyanidins, anthocyanins, and phenolic acids but low in flavonols [15]. This observation concerning the phenolic content was also confirmed by our results (Table 2).

Application of different fruit processing and juice extraction techniques on the one hand, the time and period of harvesting and level of ripening on the other, may cause variations in the phenolic content of chokeberry fruits and fruit products, including pomace and juice [4,39,48,49,50]. However, chokeberry products, including juices, remain very rich in phenolic compounds and high in antioxidant activity [51]. An important fact is that warm and dry weather correlates with higher content of phenolic compounds [52]. As was suggested already by other authors, the warm climate in Bulgaria may positively affect the polyphenolic content of local chokeberry fruits and juices [39]. In our study, all assessed AM juices available on the Bulgarian food market revealed a high content of anthocyanins, proanthocyanidins, and hydroxycinnamic acids. We found the highest anthocyanin content compared to other tested samples in juice 3. This might be explained by the period of harvesting, as the anthocyanins double after the fifth week of harvesting [4], or by the technology of juice production [49].

In our comparative study, we found cyaniding-3-O-galactoside to be the main anthocyanin, followed by cyaniding-3-O-arabinoside. A similar observation regarding the presence of cyanidin-3-O-glucoside and cyanidin-3-O-xyloside in chokeberry fruits and juices was reported by other authors [2,15]. In accordance with our previous results [53], we found that the commercially available juices contain mainly epicatechine and ten times less catechin. There are data for a notably high degree of proanthocyanidin polymerization in chokeberry juices and extracts [54], which may explain the higher levels of their mono- and oligomeric forms [15,39]. In the tested juices, the content of anthocyanins and proanthocyanidins varied from 8.1% to 19.9% and from 33.7% to 29.3%, respectively (Figure 1). The observed high variation in anthocyanin content between tested juices might be due to the higher content of anthocyanins in juice 3 and especially of cyaniding-3-O-galactoside and arabinoside.

Most of the literature concerns the content of *trans*-resveratrol in chokeberry wine and not resveratrol and resveratrol-3-glucoside levels in chokeberry fruit juice [55]. Recently, we found a low concentration of trans-resveratrol-3-O-glucoside in the tested juices.

Herrmann et al. [56] reported that hydroxycinnamic acids are the most abundant phenolic acids in plants. They represent about 50% of all detected polyphenols in our samples and vary from 45.3% (juice 3) to 51.4% (juice 2) and to 51.5% (juice 1). Neochlorogenic and chlorogenic acids are dominating among all detected phenolic acids in our samples. Chlorogenic acid and 3-O-p-coumaroylquinic acid were previously reported in chokeberry fruit juice [53]; coumaric and caffeic acid glucosides were found in chokeberry fruits [57] and were also found in our samples. We have newly reported the presence of caffeic acid-O-galactoside and 4-O-p-coumaroylquinic acid in chokeberry juice. Quinic and ferulic acid were also found in chokeberry juices [39]. We recently reported the presence of ferulic acid glucoside and galactoside and feruloylquinic acid.

Other authors showed that flavonol content in chokeberry fruit juice and extracts is relatively low and represents only 1.3% of phenolic content [15]. In agreement with these data, in our samples, we found flavonol content between 3% and 3.3% of all analyzed polyphenols, mainly presented by quercetin glycosides. Flavonol glycosides such as quercetin-3-O-rhamnosyl-galactoside and glucoside, quercetin-3-O-galactoside and glucoside [58], quercetin-3-O-arabinoside and 3-O-xyloside [59,60], kaempferol-3-O-glucoside [59], and 3-O-galactoside [60] found in our samples, were reported in AM fruits also by other authors. In addition, we established the presence of kaempferol-3-O-rhamnosyl-galactoside and glucoside, kaempferol-3-O-arabinoside and xyloside in our commercially available chokeberry juices.

Phenolic content may vary in chokeberry fruit juice even within a year of harvesting and production [4,39]. Even standardized plant extracts need periodical detailed phytochemical analysis. Therefore, the variations in chokeberry fruit juices’ phytochemical composition might be a prerequisite for the differences in their biological effects. Thus, updating the information for the types of bioactive ingredients, their quantity and quality may be useful for the evaluation of the health benefits of chokeberry juices as functional foods.

The novelty of our study is that, for the first time, a detailed comparative analysis was performed regarding the phytochemical composition of locally produced and commercially available AM fruit juices. Moreover, some of the detected phytocompounds were reported for the first time. The obtained phytochemical data could be used as a basis for the evaluation of Bulgarian AM fruit juices as functional foods.

A limitation of the current study is that it provides a snapshot regarding the phytochemical composition of locally produced AM fruit juices. It would be useful to seek relationships between juice phytochemical composition and some growth conditions, such as climate changes during the years and soil characteristics. The future objective would be a longitudinal study on the composition and quality of fruit juices produced by the same companies.

## 4. Materials and Methods

### 4.1. Plant Material

In the recent study, we analyzed samples from three selected juices from *Aronia melanocarpa* L. fresh fruits harvested locally, produced by three different Bulgarian companies, and available on the food market. All tested juices were produced from cultivated Aronia melanocarpa L. plants. There is information for the grown conditions only for the plants used for the production of juices 2 and 3. There is no available information regarding soil agrochemical characteristics.

Information regarding the analyzed samples and the technology of their production is presented in Table 3.

### 4.2. Phytochemical Analysis

Phytochemical analysis was described in more detail in our previous study [61].

For each juice, we used six bottles/bags and tested five parallel samples of each.

#### 4.2.1. Extraction

In brief, the sample preparation includes solid phase extraction (SPE) on Discovery^®^ DSC-18 column (5 g, 20 mL, Sigma-Aldrich Co. LLC, St. Louis, MO, USA). Filtered (0.45 µm PTFE filter, Waters, Milford, MA, USA) juice samples were loaded onto the SPE columns, and the anthocyanin fraction (C) was eluted 12 mL acetonitrile containing 0.1% (*v*/*v*) formic acid; the fraction containing phenolic acids, flavanols, and flavonols (B) was eluted with 12 mL ethyl acetate. The polar fraction (A) was eluted with 12 mL of water containing 0.2% (*v*/*v*) formic acid. The dry residues of all eluates were obtained after evaporation under reduced pressure at a temperature below 40 °C.

#### 4.2.2. GC-MS Analysis of Fraction A

A total of 0.2 mL of fraction A was lyophilized (6 h, −20 °C). The derivatization step was performed using methoxyamine hydrochloride (300.0 µL, 20.0 mg/mL in pyridine) on Thermo-Shaker TS-100 (1 h/70 °C/300 rpm). A total of 100.0 µL N,O-Bis (trimethylsilyl)trifluoroacetamide (BSTFA) were added to the mixture under heating (40 min/70 °C 300 rpm; Thermoshaker, Analytik Jena AG, Jena, Germany) and 1.0 µL of the solution was subjected to GC-MS analysis (Agilent GC 7890, Agilent MD 5975; column HP-5: length 30 m, diameter 0.32 mm, film thickness 0.25 μm). A temperature gradient was used for optimal separation: initial 100 °C for 2 min; ramp up to 180 °C with 15 °C/min for 1 min; ramp up to 300 °C with 5 °C/min for 10 min. Injector and detector temperatures were 250 °C. Helium was used as a carrier gas, with a rate of 1.0 mL/min. The MS scanning range was 50–550 m/z.

#### 4.2.3. LC-MS/MS Analysis of Fractions B and C

Fractions B and C were analyzed by LC-PDA-ESI-MS/in negative ESI mode for fraction B and in positive ESI mode for fraction C, as previously described [61].

The dry residues of fractions B and C were dissolved in 200 μL methanol:formic acid, (99:1 *v*/*v*), and 2 µL of the filtered solution (0.22 µm PTFE filter) were subjected to LC-PDA-ESI-MS/MS analysis.

Mass-spectrometric analysis was done on LTQ Orbitrap mass spectrometer (Thermo Scientific, Hemel Hempstead, UK) equipped with an ESI source. Operation parameters: source voltage—4 kV; sheath, auxiliary, and sweep gas—20, 10, and 2 arbitrary units, respectively; capillary temperature—275 °C. The analysis was done in full scan mode, the resolution 30,000 at m/z 400, and data-dependent MS/MS events were acquired at a resolving power of 15,000. Ions with lower intensity were analyzed in MS2 mode, resolution power of 15,000 at m/z 400, isolation width 100 amu. Precursor fragmentation was performed at collision energy 30 V, activation time 10 ms. The mass range in FTMS mode was from m/z 100 to 1000. XCalibur software v2.0.7 (Thermo Fisher Scientific, Hemel Hempstead, UK) was used for data analyses.

Chromatographic analysis was done on Accela chromatograph (Thermo Scientific, Waltham, MA, USA). Optimal separation was achieved on Kinetex C18 column (100 Å, 2.6 μm, 150 × 2.1 mm, Phenomenex Inc, Torrance, CA, USA) in a gradient elution mode: A—water/0.1% formic acid; B—acetonitrile; 0 min, 10% B; 1 min, 10% B; 15 min, 30% B; 22 min, 50% B; 28 min, 100% B; 34 min, 100% B, 36 min, 10% B; flow rate 0.3 mL/min.

#### 4.2.4. Qualitative and Quantitative Analyses

The identification of compounds in fraction A was carried out by two approaches: (1) by comparison of the retention times and Kovach indexes (RI) with the same parameters of corresponding pure standards; and (2) by using Golm Metabolome Database libraries (http://csbdb.mpimp-golm.mpg.de/csbdb/gmd/gmd.html, accessed on 30 August 2021) and NIST’08 (National Institute of Standards and Technology, Gaithersburg, MD, USA). Using the first approach, we confirmed the presence of fifteen phenolic compounds in our samples. The second approach was used for the identification of the remaining compounds.

Phenolics in fractions B and C were quantified by the external standard method as previously described [62].

### 4.3. Statistical Analysis

For statistical data analysis, we used GraphPad Prism v7.0 software (GraphPad Software, Inc.; La Jolla, CA, USA). The values of *p* < 0.05 were considered as significant. Data were presented as mean ± SD. All analyses were performed in triplicates.

## 5. Conclusions

This study provides for the first time a detailed comparative phytochemical analysis of local commercially available chokeberry juices and reports newly detected amino acids, organic acids, sugar acids, fatty acids and esters, and polyphenols. Considering the results, we may conclude that the juice acquisition techniques, pasteurization, and expected differences in plant growth conditions may cause differences in the phytochemical composition of chokeberry juices. We demonstrated a diversity of the active substances in bioactive foods marketed as “same”; therefore, the standardized therapeutic effect could be expected only by the utilization of food supplements with guaranteed constant content. Having in mind the well-known health effects of the detected bioactive compound, we may suggest that commercial black chokeberry juices are a valuable source of health-promoting phenolic acids, proanthocyanidins, and anthocyanins, thus characterizing them as a functional food.

## Figures and Tables

**Table 1:** Polar phytochemicals identified in fraction A of *Aronia melanocarpa* L. fruit juice using GC-MS. The concentration is given in µg/mL. Results are presented as mean ± standard deviation.

Compound	AM Juice 1 Content, µg/mL	AM Juice 2 Content, µg/mL	AM Juice 3 Content, µg/mL
**Amino Acids**			
*L-Valine*	3.24 ± 0.06	3.41 ± 0.05	3.62 ± 0.08
*L-Leucine*	8.63 ± 0.17	9.10 ± 0.14	9.46 ± 0.15
*L-Isoleucine*	9.08 ± 0.18	9.57 ± 0.15	9.96 ± 0.15
*L-Threonine*	4.16 ± 0.08	4.39 ± 0.07	4.56 ± 0.07
*L-Phenylalanine*	10.98 ± 0.22	11.57 ± 0.18	12.04 ± 0.19
*L-Lysine*	4.68 ± 0.09	4.93 ± 0.08	5.13 ± 0.08
L-Proline	21.43 ± 0.42	22.59 ± 0.35	23.50 ± 0.36
Glycine	4.05 ± 0.08	4.27 ± 0.07	4.44 ± 0.07
Serine	2.77 ± 0.06	2.92 ± 0.05	3.04 ± 0.05
L-Aspartic acid	17.49 ± 0.34	18.42 ± 0.28	19.17 ± 0.29
L-Asparagine	6.63 ± 0.13	6.98 ± 0.11	7.27 ± 0.11
L-Glutamic acid	1.44 ± 0.03	1.52 ± 0.02	1.58 ± 0.02
L-Tyrosine	2.85 ± 0.06	3.00 ± 0.05	3.12 ± 0.05
*Total essential AAs*	40.78 ± 0.79	42.97 ± 0.67	44.76 ± 0.61
Total non-essential AAs	56.66 ± 1.12	59.71 ± 0.92	62.12 ± 0.96
Total AAs	97.44 ± 1.90	102.67 ± 1.58	106.88 ± 1.56
**Organic Acids**			
Succinic acid	13.54 ± 0.27	14.26 ± 0.22	14.84 ± 0.23
Fumaric acid	7.08 ± 0.14	7.46 ± 0.11	7.76 ± 0.12
Malic acid	9.88 ± 0.20	10.41 ± 0.16	10.83 ± 0.17
Pyroglutamic acid (5-oxoproline)	36.03 ± 0.71	37.96 ± 0.59	39.50 ± 0.61
4-Aminobutyric acid	6.10 ± 0.12	6.42 ± 0.10	6.68 ± 0.11
2-Hydroxyglutaric acid	4.36 ± 0.09	4.59 ± 0.07	4.78 ± 0.07
2-Ketoglutaric acid	8.60 ± 0.17	9.06 ± 0.14	9.42 ± 0.15
Phenylpyruvic acid	2.33 ± 0.05	2.46 ± 0.04	2.56 ± 0.04
2,3-Dihydroxybutanedioic acid	11.23 ± 0.23	11.84 ± 0.19	12.32 ± 0.19
Isocitric acid	19.41 ± 0.38	20.45 ± 0.31	21.28 ± 0.33
Total organic acids	118.55 ± 2.34	124.91 ± 1.93	129.96 ± 2.01
**Sugar Alcohols**			
Glycerol	38.69 ± 0.76	40.78 ± 0.63	42.42 ± 0.66
Digalactosylglycerol	7.48 ± 0.15	7.88 ± 0.12	8.20 ± 0.13
Threitol	8.20 ± 0.17	8.65 ± 0.13	8.99 ± 0.14
Erythreol	2.23 ± 0.05	2.35 ± 0.04	2.45 ± 0.04
Xylitol	4.50 ± 0.09	4.75 ± 0.08	4.94 ± 0.08
Arabinitol	37.12 ± 0.73	39.11 ± 0.61	40.69 ± 0.63
L-Glycerol-3-phosphate	18.99 ± 0.37	20.00 ± 0.31	20.82 ± 0.33
Manitol	3.19 ± 0.06	3.36 ± 0.05	3.50 ± 0.06
Sorbitol	52.77 ± 1.04	55.61 ± 0.86	57.86 ± 0.90
Galactitol	2.05 ± 0.04	2.16 ± 0.04	2.25 ± 0.03
Myo-inositol	7.19 ± 0.14	7.57 ± 0.12	7.88 ± 0.12
Galactosylglycerol	25.92 ± 1.10	28.93 ± 0.91	31.31 ± 0.95
Sorbitol-6-phosphate	46.41 ± 0.91	48.90 ± 0.76	50.87 ± 0.79
myo-Inositol-1-phosphate isomer	6.04 ± 0.12	6.37 ± 0.10	6.62 ± 0.10
myo-Inositol-2-phosphate isomer	7.96 ± 0.16	8.39 ± 0.13	8.73 ± 0.13
myo-Inositol-1-phosphate isomer	3.54 ± 0.07	3.73 ± 0.06	3.88 ± 0.06
myo-Inositol-2-phosphate isomer	7.36 ± 0.15	7.76 ± 0.12	8.07 ± 0.13
Maltitol; alpha-D-Glc-(1,4)-D-sorbitol	5.25 ± 0.11	5.54 ± 0.09	5.76 ± 0.09
Galactinol isomer; alpha-D-Gal-(1,3)-myo-Inositol	0.74 ± 0.02	0.78 ± 0.02	0.81 ± 0.02
Galactinol isomer; alpha-D-Gal-(1,3)-myo-Inositol	3.93 ± 0.08	4.14 ± 0.07	4.31 ± 0.07
Total sugar alcohols	282.09 ± 6.12	298.87 ± 5.10	312.14 ± 5.30
**Sugar acids**			
Glyceric acid	18.27 ± 0.36	19.25 ± 0.30	20.02 ± 0.31
Erithreonic acid	2.84 ± 0.06	2.99 ± 0.05	3.11 ± 0.05
Threonic acid	9.00 ± 0.18	9.49 ± 0.15	9.87 ± 0.16
Pentonic acid	8.24 ± 0.17	8.69 ± 0.13	9.04 ± 0.14
Ribonic acid	5.10 ± 0.10	5.37 ± 0.08	5.59 ± 0.09
Glucuronic acid isomer	9.09 ± 0.18	9.58 ± 0.15	9.96 ± 0.15
Galacturonic acid isomer	17.05 ± 0.34	17.97 ± 0.28	18.69 ± 0.29
Glucuronic acid isomer	13.96 ± 0.27	14.71 ± 0.22	15.30 ± 0.24
Gluconic acid isomer	1.91 ± 0.04	2.02 ± 0.03	2.10 ± 0.03
Galacturonic acid isomer	3.09 ± 0.06	3.26 ± 0.05	3.39 ± 0.06
Glucuronic acid isomer	4.15 ± 0.08	4.37 ± 0.07	4.55 ± 0.07
Galactonic acid	6.78 ± 0.14	7.15 ± 0.11	7.43 ± 0.12
Gluconic acid isomer	3.98 ± 0.08	4.19 ± 0.07	4.36 ± 0.07
Glucaric acid	16.81 ± 0.92	22.66 ± 5.21	31.32 ± 0.79
Galactaric acid	3.63 ± 0.07	3.82 ± 0.06	3.97 ± 0.06
Gluconic acid-6-phosphate	1.64 ± 0.04	1.73 ± 0.03	1.80 ± 0.03
Total sugar acids	125.54 ± 3.06	137.24 ± 4.16	150.51 ± 2.65
**Saccharides (mono-, di-, and tri-)**			
Xylose methoxyamine	6.36 ± 0.13	6.52 ± 0.21	6.97 ± 0.11
Arabinose methoxyamine	13.37 ± 0.15	14.28 ± 0.22	14.86 ± 0.23
Fructose isomer	15.33 ± 0.30	16.16 ± 0.25	16.81 ± 0.26
Fructose isomer	20.24 ± 0.40	21.33 ± 0.33	22.19 ± 0.34
Sorbose isomer	30.12 ± 0.60	31.74 ± 0.49	33.02 ± 0.51
Sorbose isomer	22.86 ± 0.45	24.10 ± 0.37	25.07 ± 0.39
Galactose isomer	37.70 ± 0.74	39.72 ± 0.61	41.33 ± 0.64
Galactose isomer	14.85 ± 0.30	15.65 ± 0.24	16.28 ± 0.25
Glucose isomer	18.58 ± 0.37	19.58 ± 0.31	20.37 ± 0.32
Glucose isomer	14.56 ± 0.29	15.34 ± 0.24	15.96 ± 0.25
Fructose-6-phosphate isomer	17.35 ± 0.34	18.28 ± 0.28	19.02 ± 0.29
Mannose-6-phosphate isomer	3.71 ± 0.08	3.91 ± 0.06	4.07 ± 0.06
Galactose-6-phosphate isomer	20.12 ± 0.40	21.21 ± 0.33	22.06 ± 0.34
Glucose-6-phosphate isomer	32.42 ± 0.64	34.17 ± 0.53	35.54 ± 0.55
Fructose-6-phosphate isomer	6.22 ± 0.13	6.56 ± 0.10	6.82 ± 0.11
Galactose-6-phosphate isomer	3.56 ± 0.07	3.75 ± 0.06	3.90 ± 0.06
Glucose-6-phosphate isomer	4.84 ± 0.10	5.10 ± 0.08	5.31 ± 0.08
Sucrose isomer; alpha-D-Glc-(1,2)-beta-D-Fru isomer	26.57 ± 0.52	28.00 ± 0.43	29.13 ± 0.45
Trehalose; alpha-D-Glc-(1,1)-alpha-D-Glc isomer	10.82 ± 0.21	11.40 ± 0.18	11.86 ± 0.18
Melibiose isomer; alpha-D-Gal-(1,6)-D-Glc isomer	19.91 ± 0.40	20.98 ± 0.32	21.83 ± 0.34
Melibiose isomer; alpha-D-Gal-(1,6)-D-Glc isomer	20.14 ± 0.40	21.23 ± 0.33	22.09 ± 0.34
Sucrose isomer; alpha-D-Glc-(1,2)-beta-D-Fru isomer	22.02 ± 0.44	23.20 ± 0.36	24.14 ± 0.38
Trehalose; alpha-D-Glc-(1,1)-alpha-D-Glc isomer	17.28 ± 0.34	18.20 ± 0.28	18.94 ± 0.29
Raffinose; alpha-D-Gal-(1,6)-alpha-D-Glc-(1,2)-beta-D-Fru isomer	13.83 ± 0.27	14.57 ± 0.23	15.16 ± 0.24
Raffinose; alpha-D-Gal-(1,6)-alpha-D-Glc-(1,2)-beta-D-Fru isomer	27.44 ± 0.54	28.91 ± 0.45	30.08 ± 0.47
Total saccharides	440.21 ± 8.38	463.89 ± 6.90	482.82 ± 7.48
**Saturated, unsaturated acids and esters**			
9-(E)-Hexadecenoic acid	9.13 ± 0.18	9.61 ± 0.15	10.00 ± 0.15
9-(Z)-Hexadecenoic acid	7.03 ± 0.14	7.41 ± 0.12	7.71 ± 0.12
Heptadecanoic acid	8.10 ± 0.16	8.54 ± 0.13	8.88 ± 0.14
Hexadecatrienoic acid	5.19 ± 0.11	5.47 ± 0.08	5.70 ± 0.09
Hexadecanoic acid (Palmitic acid)	7.03 ± 0.14	7.41 ± 0.11	7.71 ± 0.12
Heptadecanoic acid	6.49 ± 0.13	6.83 ± 0.11	7.11 ± 0.11
9,12-(Z,Z)-Octadecadienoic acid (Linoleic acid)	10.38 ± 0.21	10.94 ± 0.17	11.39 ± 0.18
9,12,15-(Z,Z,Z)-Octadecatrienoic acid (Linolenic acid)	9.01 ± 0.18	9.50 ± 0.15	9.88 ± 0.15
Nonadecanoic acid	2.50 ± 0.05	2.64 ± 0.04	2.75 ± 0.04
Octadecanoic acid (Stearic acid)	11.91 ± 0.23	12.55 ± 0.19	13.05 ± 0.20
(2E,4E)-2,4-Octadecadienoic acid	16.76 ± 0.33	17.66 ± 0.27	18.38 ± 0.29
1-Monopalmitin	14.79 ± 0.29	15.58 ± 0.24	16.21 ± 0.25
Monooctadecanoylglycerol	9.24 ± 0.18	9.73 ± 0.15	10.13 ± 0.16
beta-Sitosterol	16.31 ± 0.32	17.18 ± 0.26	17.88 ± 0.28
Total saturated, unsaturated acids and esters	141.34 ± 2.77	148.95 ± 2.31	154.96 ± 2.40

AM—*Aronia melanocarpa* L.; AAs—amino acids. All metabolites are trimethylsilyl derivatives. Essential AAs are given in *italic*. Additional data regarding chromatographic parameters and total ion chromatogram of tested polar compounds are given in Table S1 and Figure S1, respectively.

**Table 2:** Polyphenolics identified in fractions B and C of the SE FAE using LC-PDA-ESI-MS/MS. The concentrations are given in µg/mL. Results are presented as mean ± standard deviation.

Compound	AM Juice 1 µg/mL	AM Juice 2 µg/mL	AM Juice 3 µg/mL
**Anthocyanins**			
Cyanidin-3-O-galactoside (idaein)	278.91 ± 6.56	262.69 ± 8.78	735.80 ± 17.41
Cyanidin-3-O-glucoside (chrysanthemin)	23.04 ± 0.61	25.62 ± 0.99	55.34 ± 1.84
Cyanidin-3-O-arabinoside	60.98 ± 2.81	60.69 ± 1.82	228.72 ± 7.53
Cyanidin-3-O-xyloside	10.91 ± 0.39	10.79 ± 0.52	30.88 ± 2.1
Total anthocyanins	373.84 ± 9.73	359.80 ± 10.46	1050.75 ± 24.86
**Proanthocyanidin monomers**			
Catechin	29.64 ± 2.42	34.60 ± 3.28	23.37 ± 1.02
Epicatechin	239.17 ± 3.05	269.47 ± 17.35	237.40 ± 3.75
Total proanthocyanidin monomers	268.81 ± 2.89	304.07 ± 14.69	260.77 ± 3.01
**Proanthocyanidin dimers**			
EC→EC(1)	128.33 ± 2.71	131.72 ± 4.09	137.45 ± 1.37
EC→EC(2)	126.71 ± 2.68	130.03 ± 4.00	135.72 ± 1.35
EC→EC(3)	142.15 ± 3.01	145.89 ± 4.50	152.25 ± 1.52
EC→EC(4)	118.22 ± 2.50	121.35 ± 3.76	126.63 ± 1.26
Total proanthocyanidin dimers	515.41 ± 10.90	529.00 ± 16.35	552.05 ± 5.49
**Proanthocyanidin trimers**			
EC→EC→EC (1)	168.32 ± 3.34	172.63 ± 5.35	180.13 ± 1.80
EC→EC→EC (2)	181.06 ± 3.59	185.69 ± 5.76	193.76 ± 1.93
EC→EC→EC (3)	148.67 ± 2.95	152.46 ± 4.73	159.09 ± 1.59
EC→EC→EC (4)	186.36 ± 3.69	191.12 ± 5.93	199.43 ± 1.99
Total proanthocyanidin trimers	684.41 ± 13.56	701.90 ± 21.76	732.42 ± 7.29
**Stilbenes**			
*trans*-Resveratrol-3-O-glucoside	39.80 ± 1.63	40.71 ± 1.42	44.37 ± 1.79
**Cyclohexanecarboxylic acid**			
Quinic acid	81.74 ± 2.27	82.66 ± 1.55	84.95 ± 0.42
**Hydroxycinnamic acids**			
3-O-Caffeoylquinic acid (chlorogenic acid)	423.08 ± 7.35	432.49 ± 13.41	451.29 ± 4.49
Caffeic acid-O-galactoside	73.66 ± 1.28	75.29 ± 2.34	78.57 ± 0.78
Caffeic acid-O-glucoside	55.71 ± 0.97	56.94 ± 1.76	59.42 ± 0.59
5-O-Caffeoylquinic acid (neochlorogenic acid)	676.03 ± 11.75	691.05 ± 21.43	721.10 ± 7.18
p-Coumaric acid-O-glucoside	176.36 ± 3.07	180.27 ± 5.59	188.11 ± 1.88
3-O-p-Coumaroylquinic acid	298.04 ± 5.18	304.67 ± 9.45	317.92 ± 3.17
Feruloylquinic acid	185.72 ± 3.23	189.85 ± 5.89	198.11 ± 1.97
4-O-p-Coumaroylquinic acid	164.01 ± 2.85	167.66 ± 5.20	174.95 ± 1.74
Ferulic acid-O-galactoside	98.23 ± 1.71	100.41 ± 3.11	104.78 ± 1.04
Ferulic acid-O-glucoside	91.22 ± 1.58	93.25 ± 2.89	97.30 ± 0.97
Total hydroxycinnamic acids	2242.06 ± 38.98	2291.88 ± 71.06	2391.55 ± 23.80
**Flavonol glycosides**			
Quercetin-3-O-rhamnosyl-galactoside	19.07 ± 0.33	19.70 ± 0.61	20.55 ± 0.20
Quercetin-3-O-galactoside (hyperoside)	21.77 ± 0.38	22.48 ± 0.70	23.45 ± 0.23
Kaempferol-3-O-galactoside	8.32 ± 0.15	8.59 ± 0.27	8.96 ± 0.09
Quercetin-3-O-rhamnosyl-glucoside	15.19 ± 0.27	15.69 ± 0.49	16.36 ± 0.16
Quercetin-3-O-glucoside (isoquercetin)	17.02 ± 0.30	17.57 ± 0.55	18.33 ± 0.18
Kaempferol-3-O-glucoside (astragalin)	7.42 ± 0.13	7.66 ± 0.24	7.99 ± 0.08
Quercetin-3-O-arabinoside (guaiaverin)	12.51 ± 0.22	12.93 ± 0.40	13.48 ± 0.13
Quercetin-3-O-xyloside	10.42 ± 0.18	10.76 ± 0.33	11.23 ± 0.11
Kaempferol-3-O-rhamnosyl-galactoside	9.34 ± 0.17	9.65 ± 0.30	10.06 ± 0.10
Kaempferol-3-O-rhamnosyl-glucoside	6.83 ± 0.12	7.05 ± 0.22	7.36 ± 0.07
Kaempferol-3-O-arabinoside	8.32 ± 0.14	8.59 ± 0.26	8.96 ± 0.09
Kaempferol-3-O-xyloside	9.55 ± 0.17	9.86 ± 0.31	10.29 ± 0.10
Total flavonol glycosides	145.75 ± 2.53	150.52 ± 4.67	157.02 ± 1.53
Total analyzed polyphenols	4351.83 ± 75.38	4460.53 ± 136.67	5273.87 ± 63.16

EC—epicatechin; AM—*Aronia melanocarpa* L. Additional data regarding precursor ion and fragment ion mass-to-charge ratios (m/z) of the analyzed polyphenols are given in Table S2. Representative LC-PDA-ESI-MS/MS chromatograms of detected polyphenols are given in Figure S2 (anthocyanins), Figure S3 (proanthocyanidin monomers), Figure S4 (proanthocyanidin di- and trimers), Figure S5 (stilbenes), Figure S6 (hydroxycinnamic acids), Figure S7 (hydroxycinnamic acids), Figures S8–S11 (flavonols).

**Table 3:** Characteristics of analyzed samples from 3 juices made by *Aronia melanocarpa* L. fresh fruits.

Characteristic	Juice 1	Juice 2	Juice 3
Organic fruits/Bio product label	N/A	yes	yes
Source of fruits/region in Bulgaria	N/A	Central Stara Planina mountain, near Kapinovski Monastery temperate-continental climate with pronounced mountain influence; latitude: 42.978677; longitude: 25.747620; altitude 203 m.	Central Stara Planina mountain, near city of Troyan climate: temperate-continental climate; latitude: 42.883, longitude: 24.717; altitude 446 m.
Processing	immediately after harvest cold press pasteurization	immediately after harvest cold press pasteurization	immediately after harvest cold press pasteurization
Additives	NO sugars, NO preservatives, NO additives	NO sugars, NO preservatives, NO additives	NO sugars, NO preservatives, NO additives
Package	250 mL glass bottle	1.5 L bag in box	270 mL glass bottle

## Data Availability

The data presented in this study are available in the article.

## Supplementary Materials

The following supporting information can be downloaded at: https://www.mdpi.com/article/10.3390/plants11131655/s1, Figure S1: Representative chromatogram of analyzed polar compounds (fraction A) by GC-MS technique, Figure S2: Representative LC-PDA-ESI-MS/MS chromatogram of *Aronia melanocarpa* L. fruit anthocyanins (1-Cyanidin-3-O-Galactoside, 2-Cyanidin-3-O-Glucoside, 3-Cyanidin-3-O-Arabinoside, 4-Cyanidin-3-O-Xyloside), Figure S3: Representative LC-PDA-ESI-MS/MS chromatogram of *Aronia melanocarpa* L. fruit proanthocyanidin monomers, Figure S4: Representative LC-PDA-ESI-MS/MS chromatogram of *Aronia melanocarpa* L. fruit pro-anthocyanidin di- and trimers, Figure S5: Representative LC-PDA-ESI-MS/MS chromatogram of *Aronia melanocarpa* L. fruit stilbenes, Figure S6: Representative LC-PDA-ESI-MS/MS chromatogram of *Aronia melanocarpa* L. fruit hydroxycinnamic acids (1–3-O-Caffeoylquinic acid, 2–Caffeic ac-id-O-galactoside, 3–Caffeic acid-O-glucoside, 4–5-O-Caffeoylquinic acid, 5–p-Coumaric ac-id-O-glucoside, 6–3-O-p-Coumaroylquinic acid, 7–Feruloylquinic acid; 8–4 -O-p-Coumaroylquinic acid; 9–Ferulic acid-O-galactoside; 10–Ferulic acid-O-glucoside), Figure S7: Representative LC-PDA-ESI-MS/MS chromatogram of *Aronia melanocarpa* L. fruit flavonols (1-Kaempferol-3-O-arabinoside, 2-Kaempferol-3-O-xyloside), Figure S8: Representative LC-PDA-ESI-MS/MS chromatogram of *Aronia melanocarpa* L. fruit flavonols (1-Quercetin-3-O-galactoside, 2-Quercetin-3-O-glucoside), Figure S9: Representative LC-PDA-ESI-MS/MS chromatogram of *Aronia melanocarpa* L. fruit flavonols (1-Kaempferol-3-O-rhamnosyl-galactoside, 2-Kaempferol-3-O-rhamnosyl-glucoside), Figure S10: Representative LC-PDA-ESI-MS/MS chromatogram of *Aronia melanocarpa* L. fruit flavonols (1-Quercetin-3-O-rhamnosyl-galactoside, 2-Quercetin-3-O-rhamnosyl-glucoside), Figure S11: Representative LC-PDA-ESI-MS/MS chromatogram of *Aronia melanocarpa* L. fruit flavonols (1-Kaempferol-3-O-galactoside, 2-Kaempferol-3-O-glucoside), Table S1: Relative Kovat’s reten-tion index (RI) of analyzed polar compounds (fraction A) presented in Table 1, using GC-MS technique, Table S2: Precursor ion and fragment ion mass-to-charge ratios (m/z) of the analyzed polyphenols using the LC-PDA-ESI-MS/MS technique.
