# Supplementary material for: Comparative Phytochemical Analysis of Aronia melanocarpa L. Fruit Juices on Bulgarian Market
Source: Plants (Basel). 2022 Jun 22;11(13):1655. doi: 10.3390/plants11131655 (PMC9269608; doi:10.3390/plants11131655)
Supplement: Supplementary file 1 [file plants-11-01655-s001.zip › plants-1787522-supplementary.pdf]

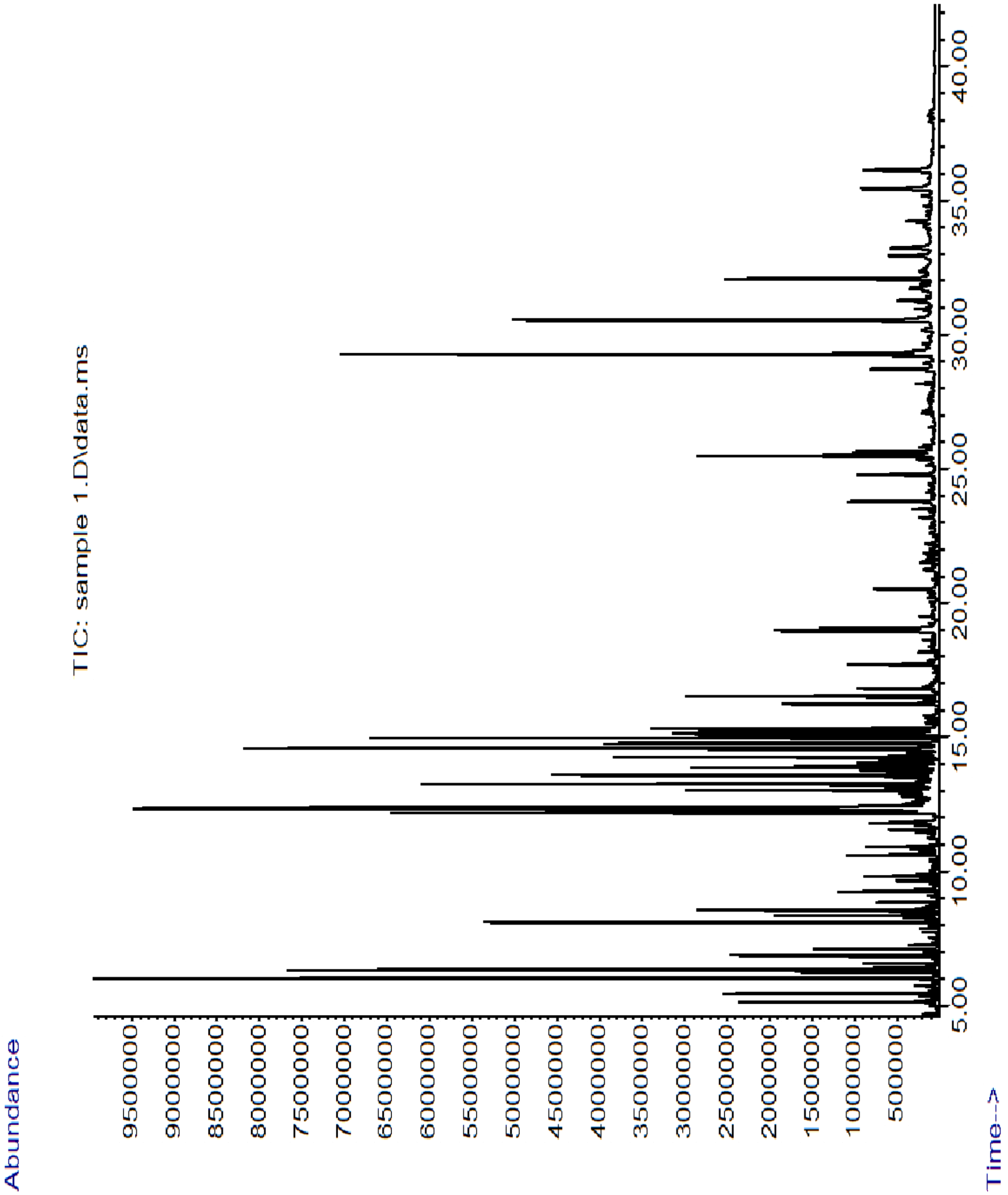

Figure S1. Representative chromatogram of analyzed polar compounds (fraction A) presented in Table 1, using GC-MS technique.

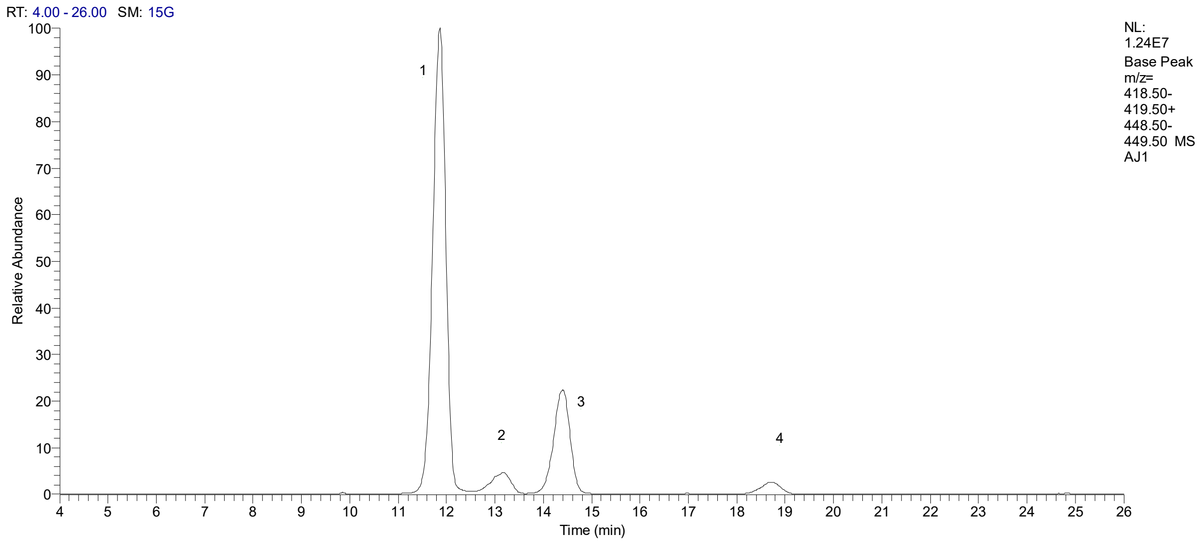

Figure S2. Representative LC-PDA-ESI-MS/MS chromatogram of *Aronia melanocarpa* L. fruit anthocyanins (1 - Cyanidin-3-O-Galactoside, 2 - Cyanidin-3-O-Glucoside, 3 - Cyanidin-3-O-Arabinoside, 4 - Cyanidin-3-O-Xyloside).

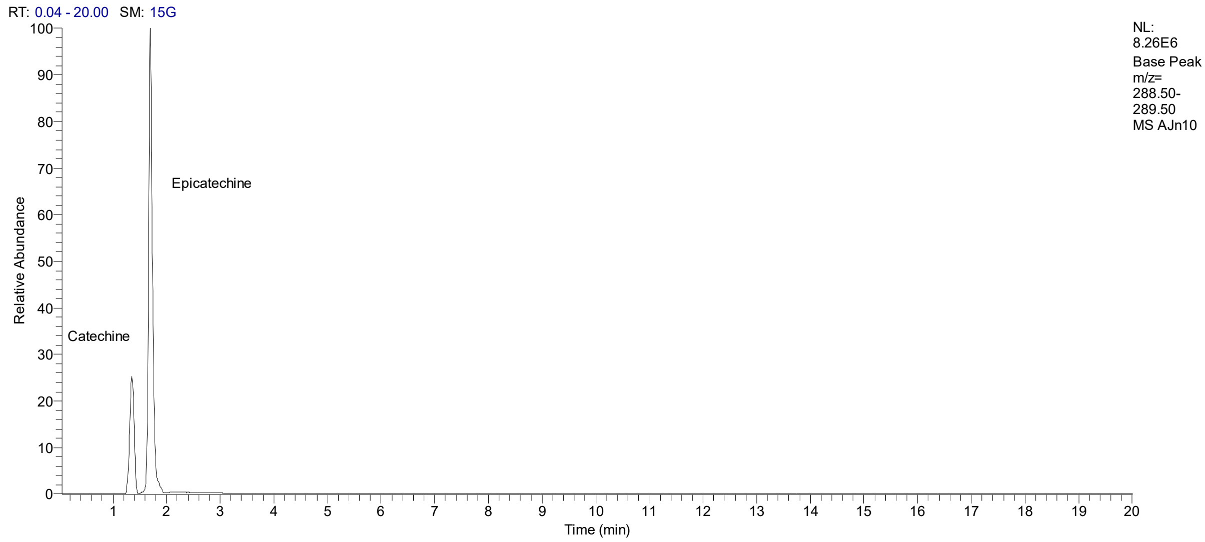

Figure S3. Representative LC-PDA-ESI-MS/MS chromatogram of *Aronia melanocarpa* L. fruit proanthocyanidin monomers.

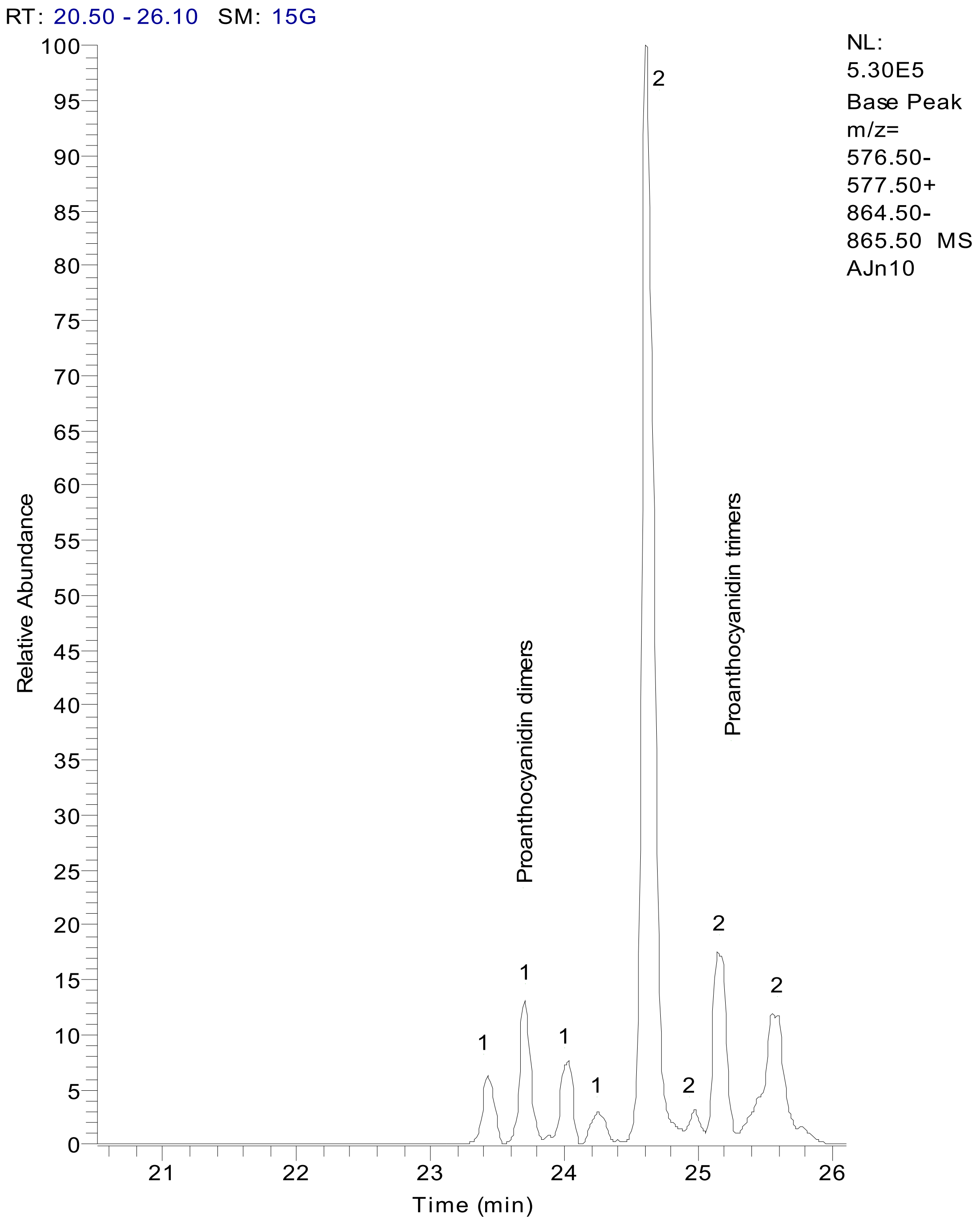

Figure S4. Representative LC-PDA-ESI-MS/MS chromatogram of *Aronia melanocarpa* L. fruit proanthocyanidin di - and trimers

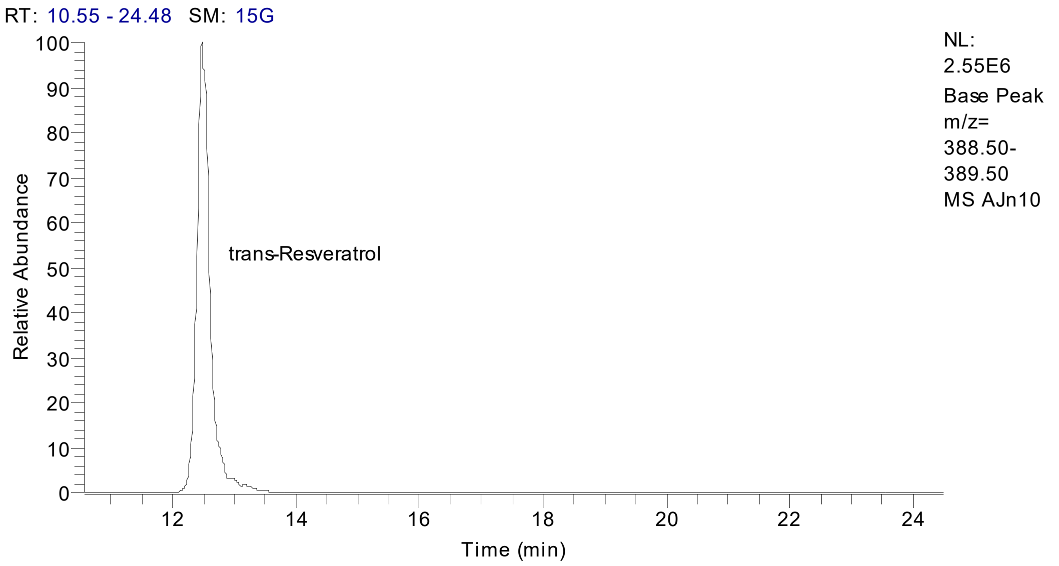

Figure S5. Representative LC-PDA-ESI-MS/MS chromatogram of *Aronia melanocarpa* L. fruit stilbenes.

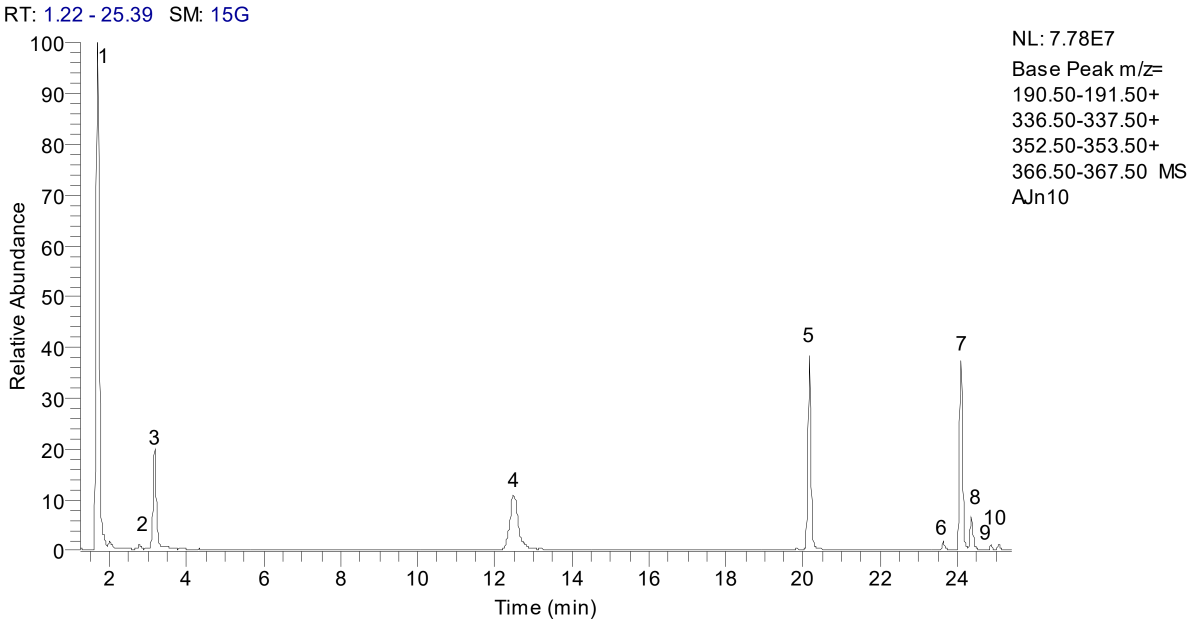

Figure S6. Representative LC-PDA-ESI-MS/MS chromatogram of *Aronia melanocarpa* L. fruit hydroxycinnamic acids (1 – 3-O-Caffeoylquinic acid, 2 – Caffeic acid-O-galactoside, 3 – Caffeic acid-O-glucoside, 4 – 5-O-Caffeoylquinic acid, 5 – p-Coumaric acid-O-glucoside, 6 – 3-O-p-Coumaroylquinic acid, 7 – Feruloylquinic acid; 8 – 4 -O-p-Coumaroylquinic acid; 9 – Ferulic acid-O-galactoside; 10 – Ferulic acid-O-glucoside);

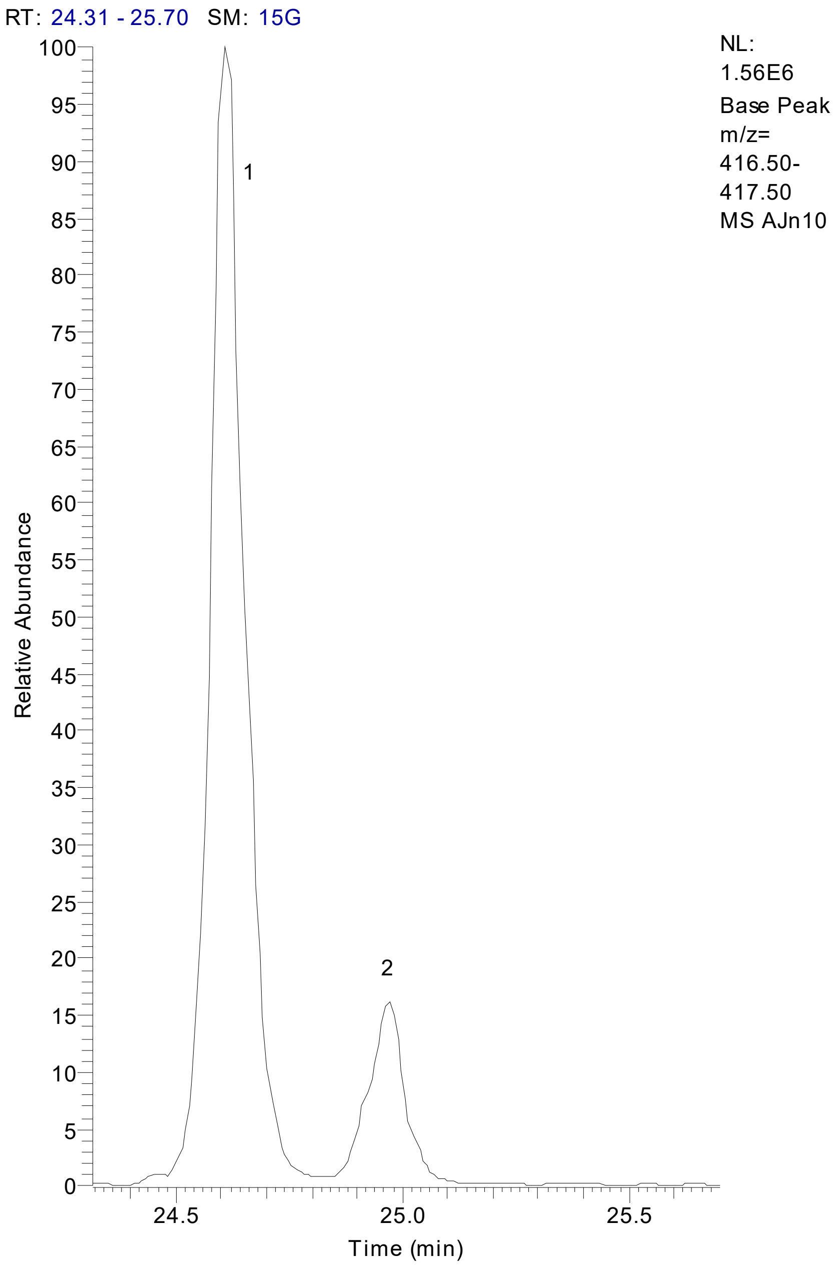

Figure S7. Representative LC-PDA-ESI-MS/MS chromatogram of *Aronia melanocarpa* L. fruit flavonols (1 - Kaempferol-3-O-arabinoside, 2 - Kaempferol-3-O-xyloside).

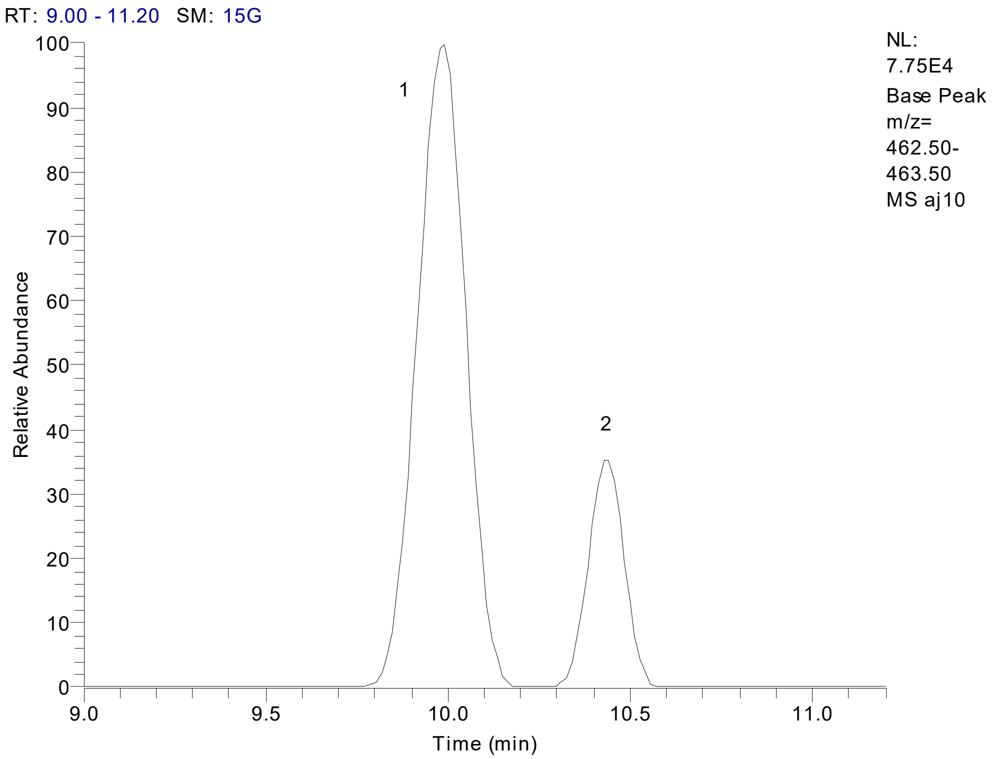

Figure S8. Representative LC-PDA-ESI-MS/MS chromatogram of *Aronia melanocarpa* L. fruit flavonols (1 - Quercetin-3-O-galactoside, 2 - Quercetin-3-O-glucoside).

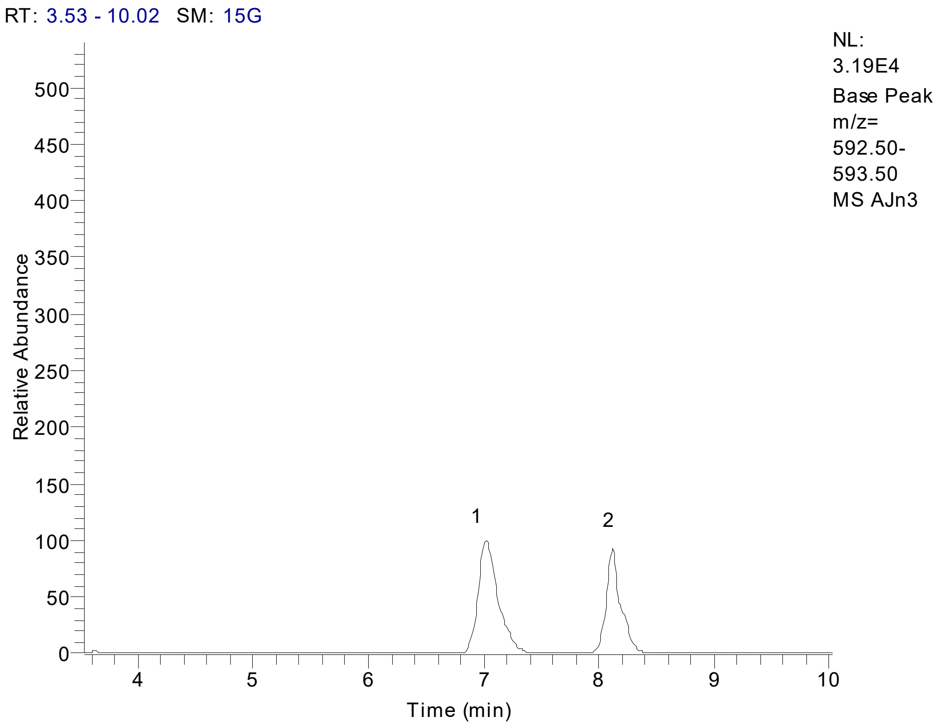

Figure S9. Representative LC-PDA-ESI-MS/MS chromatogram of *Aronia melanocarpa* L. fruit flavonols (1 - Kaempferol-3-O-rhamnosyl-galactoside, 2 - Kaempferol-3-O-rhamnosyl-glucoside).

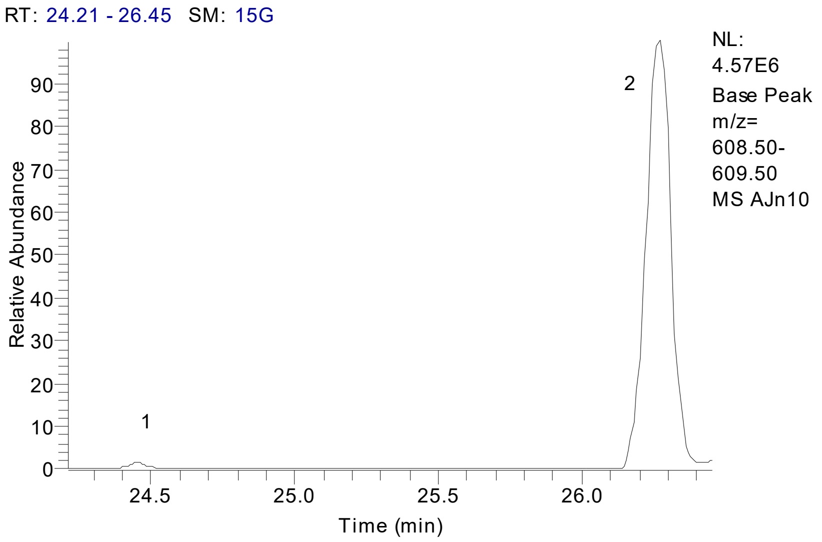

Figure S10. Representative LC-PDA-ESI-MS/MS chromatogram of *Aronia melanocarpa* L. fruit flavonols (1 - Quercetin-3-O-rhamnosyl-galactoside, 2 - Quercetin-3-O-rhamnosyl-glucoside).

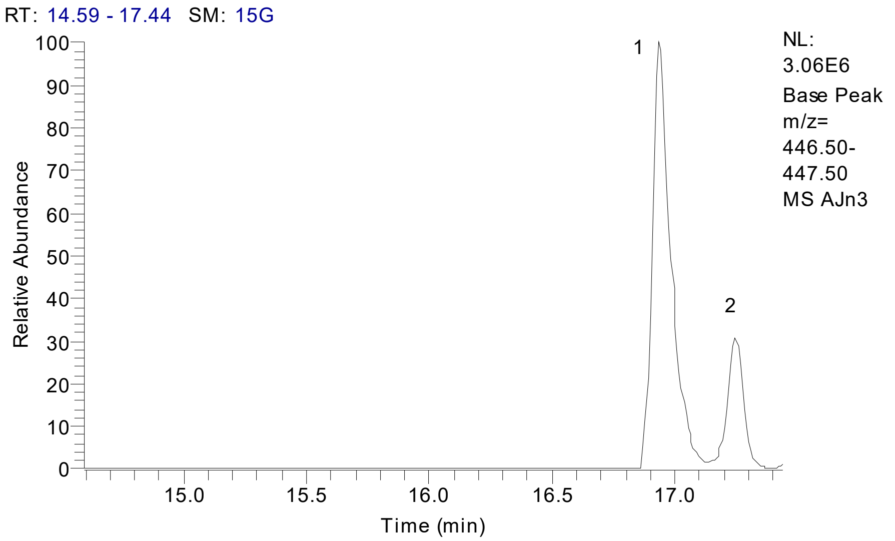

Figure S11. Representative LC-PDA-ESI-MS/MS chromatogram of *Aronia melanocarpa* L. fruit flavonols (1 - Kaempferol-3-O-galactoside, 2 - Kaempferol-3-O-glucoside).

Table S1. Relative Kovats retention index (RI) of analyzed polar compounds (fraction A) presented in Table 1, using GC-MS technique.

Compound	RI
**Amino acids**	
L-Valine (2TMS)	1222.5
L-Leucine (2TMS)	1279.9
L-Isoleucine (2TMS)	1298.4
L-Proline (2TMS)	1304.3
L-Threonine (3TMS)	1392.0
L-Phenylalanine (2TMS)	1635.4
L-lysine (3TMS)	1856.8
Glycine (3TMS)	1314.7
Serine (3TMS)	1368.4
L-Aspartic acid (3TMS)	1519.2
L-Asparagine (3TMS)	1609.2
L-Glutamic acid (3TMS)	1626.3
L-Glutamine (3TMS)	1784.5
DL-Ornithine (3TMS)	1759.6
L-Tyrosine (3TMS)	1942.2
**Organic acids**	
Succinic acid (2TMS)	1329.2
Fumaric acid (2TMS)	1357.9
Malic acid (2TMS)	1490.6

Pyroglutamic acid (2TMS)	1529.3
4-Aminobutyric acid (3TMS)	1533.6
2-Hydroxyglutaric acid (3TMS)	1576.4
2-Ketoglutaric acid methoxyamine (2TMS)	1583.5
Phenylpyruvic acid methoxamine (1TMS)	1599.9
2,3-Dihydroxybutanedioic acid (4TMS)	1640.2
Isocitric acid (4TMS)	1832.0
**Sugar acids and alcohols**	
Glycerol (3TMS)	1282.6
Digalactosylglycerol (9TMS)	3140.3
Glyceric acid (2TMS)	1341.7
Threitol (4TMS)	1502.6
Erythreol (4TMS)	1512.0
Erithreonic acid (4TMS)	1547.4
Threonic acid (4TMS)	1567.2
Xylitol (5TMS)	1699.6
Arabinitol (5TMS)	1714.1
Pentonic acid (5TMS)	1754.5
L-Glycerol-3-phosphate (4TMS)	1770.0
Ribonic acid (3TMS)	1789.6
Manitol (6TMS)	1928.6
Sorbitol (6TMS)	1933.2
Glucuronic acid methoxyamine (5TMS) isomer	1937.8
Galactitol (6TMS)	1942.0
Galacturonic acid methoxyamine (5TMS) isomer	1945.6
Glucuronic acid methoxyamine (5TMS) isomer	1951.9
Gluconic acid (6TMS) isomer	1961.8
Galacturonic acid methoxyamine (5TMS) isomer	1966.3
Glucuronic acid methoxyamine (5TMS) isomer	1977.4
Galactonic acid (6TMS)	1991.7
Gluconic acid (6TMS) isomer	2002.5
Glucaric acid (6TMS)	2013.6
Galactaric acid (6TMS)	2041.2
Myo-inositol	2090.3
Galactosylglycerol (6TMS)	2309.6
Sorbitol-6-phosphate (7TMS)	2378.4
myo-Inositol-1-phosphate (7TMS) isomer	2424.2
myo-Inositol-2-phosphate (7TMS) isomer	2431.5
Gluconic acid-6-phosphate (7TMS)	2441.2
myo-Inositol-1-phosphate (7TMS) isomer	2479.4
myo-Inositol-2-phosphate (7TMS) isomer	2485.3
Maltitol (9TMS); alpha-D-Glc-(1,4)-D-sorbitol (9TMS)	2838.5
Galactinol (9TMS) isomer; alpha-D-Gal-(1,3)-myo-Inositol (9TMS)	2950.3
Galactinol (9TMS) isomer; alpha-D-Gal-(1,3)-myo-Inositol (9TMS)	2990.2
**Saccharides (mono-, di-, and tri-)**	

Xylose methoxyamine (4TMS)	1661.2
Arabinose methoxyamine (4TMS)	1673.2
Fructose methoxyamine (5TMS) isomer	1861.9
Fructose methoxyamine (5TMS) isomer	1869.8
Sorbose methoxyamine (5TMS) isomer	1870.6
Sorbose methoxyamine (5TMS) isomer	1878.3
Galactose methoxyamine (5TMS) isomer	1885.5
Galactose methoxyamine (5TMS) isomer	1893.3
Glucose methoxyamine (5TMS) issomer	1895.2
Glucose methoxyamine (5TMS) issomer	1910.2
Fructose-6-phosphate methoxyamine (6TMS) isomer	2307.8
Mannose-6-phosphate methoxyamine (6TMS) isomer	2312.4
Galactose-6-phosphate methoxyamine (6TMS) isomer	2315.3
Glucose-6-phosphate methoxyamine (6TMS) isomer	2318.7
Fructose-6-phosphate methoxyamine (6TMS) isomer	2321.2
Galactose-6-phosphate methoxyamine (6TMS) isomer	2347.6
Glucose-6-phosphate methoxyamine (6TMS) isomer	2351.6
Sucrose (8TMS) isomer; alpha-D-Glc-(1,2)-beta-D-Fru isomer	2651.2
Trehalose (8TMS); alpha-D-Glc-(1,1)-alpha-D-Glc isomer	2750.2
Melibiose methoxyamine (8TMS) isomer; alpha-D-Gal-(1,6)-D-Glc (8TMS) isomer	2870.8
Melibiose methoxyamine (8TMS) isomer; alpha-D-Gal-(1,6)-D-Glc (8TMS) isomer	2903.4
Sucrose (8TMS) isomer; alpha-D-Glc-(1,2)-beta-D-Fru isomer	3003.3
Trehalose (8TMS); alpha-D-Glc-(1,1)-alpha-D-Glc isomer	3048.9
Raffinose (11TMS) isomer; alpha-D-Gal-(1,6)-alpha-D-Glc-(1,2)-beta-D-Fru (11TMS) isomer	3374.4
Raffinose (11TMS) isomer; alpha-D-Gal-(1,6)-alpha-D-Glc-(1,2)-beta-D-Fru (11TMS) isomer	3393.7
**Saturated, unsaturated acids and esters**	
Hexadecenoic acid (1TMS)	2022.3
9-(Z)-Hexadecenoic acid (1TMS)	2025.9
Heptadecanoic acid (1TMS)	2029.8
Hexadecatrienoic acid (1TMS)	2033.3
Hexadecanoic acid (1TMS)	2046.3
Heptadecanoic acid (1TMS)	2141.4
9,12-(Z,Z)-Octadecadienoic acid (1TMS)	2212.8
9,12,15-(Z,Z,Z)-Octadecatrienoic acid (1TMS)	2221.5
Nonadecanoic acid (1TMS)	2233.1
Octadecanoic acid (1TMS)	2243.0
Octadecadienoic acid (1TMS)	2313.5
1-Monopalmitin trimethylsilyl ether	2466.8
Monooctadecanoylglycerol (2TMS)	2832.6
beta-Sitosterol (1TMS)	3227.8

Table S2. Precursor ion and fragment ion mass to charge ratios (m/z) of analyzed polyphenols using LC-PDA-ESI-MS/MS technique.

Compound	[M+H]+ (m/z)	MS/MS ions
**Anthocyanins**		
Cyanidin-3-O-Galactoside	449	287
Cyanidin-3-O-Glucoside	449	287
Cyanidin-3-O-Arabinoside	419	287
Cyanidin-3-O-Xyloside	419	287
**Proanthocyanidin monomers**		
Catechin	289	245(100), 205(35), 179(15)
Epicatechin	289	245(100), 205(30), 179(10)
**Proanthocyanidin dimers**		
EC→EC(1)	577	559(17), 451(37), 425(100), 407(53), 299(8), 289(26), 287(8)
EC→EC(2)	577	559(57), 467(20), 451(100), 425(86), 407(59), 289(65)
EC→EC(3)	577	559(75), 533(46), 451(29), 439(67), 425(75), 407(20), 393(100), 289(29), 269 (35)
EC→EC(4)	577	559(100), 533(31), 451(21), 439(34), 425(32), 407(18), 393(35)
**Proanthocyanidin trimers**		
EC→EC→EC (1)	865	847(40), 779(51), 739(56), 713(57), 695(68), 577(89), 575(100), 449(22), 407(35), 289(27), 287(24)
EC→EC→EC (2)	865	847(38), 739(100), 713(58), 695(87), 577(64), 575(35), 451(37), 449(26), 407(30), 287(29)
EC→EC→EC (4)	865	847(18), 749(48), 695(100), 577(68), 575(31), 425(27), 407(30)
EC→EC→EC (4)	865	801(41), 789(49), 779(100), 720(70), 695(51), 577(74), 575(55)
Stilbenes		
trans-Resveratrol-3-O-glucoside	389	227 (100)
**Cyclohexanecarboxylic acid**		
Quinic acid	191	173 (5), 11 (60), 109 (5)
**Hydroxycinnamic acids**		
3-O-Caffeoylquinic acid	353	191 (100), 179 (60), 135 (20)
Caffeic acid-O-galactoside	341	179 (100), 135 (10)
Caffeic acid-O-glucoside	341	179 (100), 135 (10)
5-O-Caffeoylquinic acid	353	191 (100)

p-Coumaric acid-O-glucoside	325	163 (100), 119 (40)
3-O-p-Coumaroylquinic acid	337	191 (10), 163 (100)
Feruloylquinic acid	367	193 (20), 191 (100), 173 (20)
4-O-p-Coumaroylquinic acid	337	191 (10), 173 (100), 163 (30)
Ferulic acid-O-galactoside	355	193 (100)
Ferulic acid-O-glucoside	355	193 (100)
**Flavonol glycosides**		
Quercetin-3-O-rhamnosyl-galactoside	609	301 (100), 179, 151
Quercetin-3-O-galactoside	463	301 (100), 179, 151
Kaempferol-3-O-galactoside	447	285 (100), 257, 169, 151
Quercetin-3-O-rhamnosyl-glucoside	609	301 (100), 179, 151
Quercetin-3-O-glucoside	463	301 (100), 179, 151
Kaempferol-3-O-glucoside	447	285 (100), 257, 169, 151
Quercetin-3-O-arabinoside	433	301 (100), 179, 151
Quercetin-3-O-xyloside	433	301 (100), 179, 151
Kaempferol-3-O-rhamnosyl-galactoside	593	285 (100), 257, 169, 151
Kaempferol-3-O-rhamnosyl-glucoside	593	285 (100), 257, 169, 151
Kaempferol-3-O-arabinoside	417	285 (100), 257, 169, 151
Kaempferol-3-O-xyloside	417	285 (100), 257, 169, 151
